# Physicochemical properties of fifteen flower honey samples from five districts in Türkiye

**DOI:** 10.1038/s41598-025-30865-x

**Published:** 2025-12-05

**Authors:** Osman Ucuncu, Merve Kalafat Kul, Cemalettin Baltaci, Ayse Muslu Aykoc

**Affiliations:** 1 Macka Vocational School, Department of Pharmacy Services, Karadeniz Technical University, Trabzon, 61750 Türkiye; 2 Faculty of Engineering and Natural Sciences, Department of Food Engineering, Gümüshane University, Gümüshane, 29000 Türkiye; 3 Faculty of Arts and Design, Department of Gastronomy and Culinary Arts, Alanya University, Antalya, 07400 Türkiye

**Keywords:** Honey, Minerals, Phenolics, Antioxidant, Hydroxymethylfurfural, Biochemistry, Biological techniques, Chemistry, Environmental sciences, Plant sciences

## Abstract

This study analyzed the physicochemical characteristics, mineral composition, and antioxidant potential of fifteen floral honey samples collected from five districts of Gümüşhane, Türkiye. The average values for moisture (17.44 g/100 g), insoluble solids (0.04 g/100 g), conductivity (0.35 mS/cm), diastase activity (54.15 DN), free acidity (38.18 meq/kg), and fructose + glucose content (68.95 g/100 g) were mostly within the acceptable limits defined by national honey standards. Proline content ranged from 562.66 to 1466.15 mg/kg, while hydroxymethylfurfural (0.37–18.89 mg/kg) confirmed the freshness of all samples. Potassium, calcium, sodium, and magnesium were identified as the major mineral elements in the analyzed honey samples, with considerable variation observed among samples collected from different districts. Antioxidant activity (Total phenolic content (mg GAE/100 g): 9.21–98.25; SC₅₀ (mg/mL): 5.23–41.29) revealed a strong antioxidant potential of Gümüşhane honeys. This work provides the first detailed characterization of Gümüşhane honeys, confirming their high physicochemical quality, rich mineral profile, and notable antioxidant potential, and underscores their value as a distinctive regional product with functional and commercial significance.

## Introduction

Honey is a natural sweet product formed as bees gather nectar from flowers, convert it through enzymatic processes, and store it in honeycombs for maturation^1^. In addition to being viscous, aromatic, and sweet, it contains enzymes, proteins, vitamins, minerals, organic acids, Maillard reaction products, various bioactive compounds, and sugars, including fructose, glucose, sucrose, and maltose^2^. Honey’s aroma is influenced by volatile compounds, while amino acids and polyphenols contribute to its maturation and antioxidant properties^3–5^. Around 320 honey types reflect the unique physicochemical and sensory properties of their floral origins^6^. The assessment of honey quality is based on several factors, including electrical conductivity, sugar composition, moisture, ash content, diastase activity, free acidity, and the amount of HMF^7^. Honey quality is regulated through national and international standards, such as the Codex Alimentarius, the EU Honey Directive, and the Turkish Food Codex Honey Communiqué^7,17^.

The characteristics of honey are influenced by factors such as plant origin, environmental, and seasonal conditions, methods of beekeeping, post-harvest processing techniques, and the physiological characteristics of honeybees. For instance, the water content of honey is influenced by the origin of the nectar, the harvest season, and the level of ripeness. Meanwhile, its aromatic substances are affected by the botanical source, honeybee physiology, and climatic conditions^8^. The floral source significantly impacts the physicochemical aspects of honey, including moisture value, color, pH level, electrical conductivity, acidity, and mineral content. Conversely, parameters such as HMF content and purity are primarily related to the production process. The acidity level reflects the degree to which the honey has ripened and serves as an indicative factor of its stability and quality during storage^7^. Its primary constituents are levulose, dextrose, water, and minerals, with only trace amounts of sucrose present^9^. Levulose and dextrose rapidly enter the bloodstream, providing instant energy. Honey is also known for its blood-purifying effects and protective role against colds, coughs, and fevers. Its therapeutic effects on eye wounds and burns have also been reported^10^. The nutritional benefits of consuming honey include improved calcium fixation in bones and the potential to treat anemia and anorexia^11^. Honey also has general medicinal and healing properties, including a soothing effect, promoting digestion, and regulating stomach acid^12^. The positive effects of honey on digestion are largely linked to its content of minerals, flavonoids, and other bioactive compounds^13^. It is estimated that the global honey production amounts to 1.72 million metric tons^14^. According to the 2023 Beekeeping Products Report, the top five countries in terms of beehive numbers are India, China, Türkiye, Iran, and Ethiopia. China ranks first in global honey production, followed by Türkiye, Ethiopia, Iran, and Argentina. China leads the market with a 25% share of total production, while Türkiye holds second place with a 6% share^15^. Türkiye is an important beekeeping country with a recorded honey production of 95.492 tons in 2024. Due to its rich botanical diversity and varied ecological structure, Gümüşhane is considered a favorable region for the production of high-quality floral honeys^16^.

Although Gümüşhane is botanically diverse, studies on the quality and composition of its honeys are limited. Therefore, this study evaluated 15 floral honey samples from five districts to characterize their physicochemical properties, antioxidant capacity, and mineral and heavy metal content.

## Results and discussion

### Physicochemical composition of honey samples

The moisture content of the floral honey samples ranged from 15.60 to 20.26 g/100 g, with the highest value observed in Sample 7 and the lowest in Sample 2. According to the Turkish Food Codex. Honey Communiqué (No. 2020/7), the maximum permissible moisture content for floral honeys is 20 g/100 g, except for heather honeys derived from *Calluna vulgaris* and *Erica species*. All honey samples in this study complied with the legal moisture limits, which may reflect proper harvesting and storage practices by local beekeepers. Moisture content in honey is known to be affected by environmental humidity, premature harvesting, and the intrinsic moisture of floral sources^18^. Elevated moisture levels may increase the risk of fermentation due to osmophilic yeast activity^19,20^. Serin et al^21^. reported moisture values ranging from 15.00 to 20.40 g/100 g in flower and pine honeys collected from Western Türkiye, while Bayram et al*.*^22^ found values between 15.00 and 18.50 g/100 g in floral honeys from the Northeastern region. The findings of the present study are in line with these reports and suggest that the moisture content of Gümüşhane honeys falls within the expected and acceptable range, indicating good quality and low risk of spoilage.

Insoluble solid matter in honey, such as wax, pollen, and honeycomb residues, is a key indicator of physical contamination and is quantified to assess compliance with purity standards^19^. Significant but slight differences (*p* < 0.05) in insoluble matter were noted among samples (Table 1). All measured values were below the maximum allowable limit of 0.1 g/100 g, as defined by the Turkish Food Codex Honey Communiqué. These results are consistent with those of Özgüven et al^19^., who reported insoluble matter values ranging from 0.01 to 0.06 g/100 g in twelve floral honey samples. Furthermore, the findings of this study revealed lower insoluble matter levels compared to sunflower honeys reported by Baloš et al^23^., which showed an average value of 0.08 g/100 g.

**Table 1:** Some physical and chemical parameters of the studied honey samples.

Samples		Physical parameters		Chemical parameters				
		Moisture (g/100 g)	Water insoluble solids (g/100 g)	Electrical conductivity (mS/cm)	Diastase activity (DN)	Free acidity (meq/kg)	Proline (mg/kg)	HMF (mg/kg)
1		16.30 ± 0.56^a^	0.02 ± 0.01^a^	0.18 ± 0.08^a^	27.30 ± 1.56^a^	31.20 ± 1.41^b^	1029.79 ± 8.25^ h^	2.51 ± 0.23^b^
2	Torul	15.60 ± 0.56^a^	0.03 ± 0.01^a^	0.35 ± 0.06^a^	50.00 ± 1.41^b^	30.20 ± 1.56^b^	1466.15 ± 16.08^j^	0.51 ± 0.21^a^
3		19.50 ± 0.42^c^	0.06 ± 0.02^ab^	0.26 ± 0.02^a^	50.00 ± 1.69^b^	**53.80 ± 1.97f.**	806.01 ± 9.90^e^	0.37 ± 0.11^a^
4		17.00 ± 0.68^ab^	0.02 ± 0.01^a^	0.28 ± 0.07^a^	> 50	37.30 ± 0.99^ cd^	722.10 ± 9.34^ cd^	2.29 ± 0.50^b^
5	Şiran	15.70 ± 0.57^a^	0.02 ± 0.01^a^	0.19 ± 0.06^a^	> 50	18.60 ± 1.27^a^	656.36 ± 7.66^b^	3.32 ± 0.32^ cd^
6		17.60 ± 0.54^abc^	0.03 ± 0.01^a^	0.20 ± 0.04^a^	> 50	16.65 ± 1.27^a^	754.27 ± 7.72^d^	18.89 ± 1.50^ g^
7		**20.26 ± 0.34**^**d**^	0.08 ± 0.02^b^	**0.99 ± 0.03**^**c**^	> 50	42.30 ± 0.70^d^	850.77 ± 7.40f.	3.99 ± 0.11^ cd^
8	Kürtün	17.90 ± 0.69^abc^	0.03 ± 0.01^a^	0.80 ± 0.05^b^	> 50	46.90 ± 1.55^de^	597.62 ± 11.02^a^	1.01 ± 0.21^ab^
9		17.40 ± 0.42^abc^	0.06 ± 0.01^ab^	0.64 ± 0.03^b^	> 50	48.50 ± 1.41^e^	929.09 ± 10.36^ g^	4.58 ± 0.11^d^
10		16.60 ± 0.85^ab^	0.05 ± 0.01^ab^	0.17 ± 0.02^a^	> 50	**59.10 ± 0.57**^** g**^	751.47 ± 11.10^d^	5.17 ± 0.14^e^
11	Kelkit	16.50 ± 0.69^ab^	0.04 ± 0.01^ab^	0.17 ± 0.04^a^	> 50	31.00 ± 1.27^b^	706.71 ± 12.39^c^	5.08 ± 0.19^de^
12		16.70 ± 0.69^ab^	0.04 ± 0.01^ab^	0.24 ± 0.05^a^	> 50	33.30 ± 1.55^bc^	1164.06 ± 17.77^ı^	9.30 ± 0.21f.
13		16.20 ± 0.56^a^	0.03 ± 0.01^a^	0.20 ± 0.03^a^	25.00 ± 1.41^a^	35.45 ± 1.70^bc^	740.28 ± 10.30^ cd^	3.20 ± 0.13^ cd^
14	Köse	18.80 ± 0.71^bc^	0.03 ± 0.01^a^	0.35 ± 0.03^a^	> 50	47.20 ± 1.69^de^	562.66 ± 11.76^a^	4.45 ± 0.14^d^
15		19.60 ± 0.57^ cd^	0.04 ± 0.01^ab^	0.25 ± 0.04^a^	> 50	41.10 ± 0.70^d^	597.62 ± 10.47^a^	3.07 ± 0.12^ cd^
*Overall Mean* ± *SD*		17.44 ± 1.51	0.04 ± 0.02	0.35 ± 0.25	54.15 ± 6.75	38.18 ± 11.81	822.33 ± 238.31	4.51 ± 4.48
Maximum		20.50	0.08	1.01	60.00	59.40	1477.52	19.95
Minimum		15.20	0.02	0.12	23.59	15.80	554.34	0.29

Different letters within the same column indicate significant differences (*p* < 0.05) based on one-way ANOVA followed by Tukey’s HSD test.

*SD Standard deviation.

Bold values exceed the limits defined by national honey standards.

Conductivity of the samples varied between 0.17 and 0.99 mS/cm, with significant differences among them (*p* < 0.05; Table 1). As per Turkish and EU regulations, the upper limit is set at 0.80 mS/cm^18^. All samples complied with this limit, except for Sample 7, which recorded a value of 0.99 mS/cm. The elevated conductivity observed in this sample may be attributed to its higher mineral content, as electrical conductivity is known to increase with the mineral, protein, and organic acid levels of the floral source^24^. The values obtained in this study are in agreement with previous findings. For instance, Bayram et al*.*^22^reported values ranging from 0.28 to 0.71 mS/cm in floral honeys from Northeastern Türkiye, while Živkov Baloš et al^25^. reported values between 0.22 and 0.54 mS/cm. Although the electrical conductivity of floral honeys typically remains below 0.8 mS/cm, exceptions have been reported depending on floral source and regional factors^22^.

The free acidity values of the analyzed Gümüşhane honey samples in the current study ranged from 16.65 to 59.10 meq/kg. The free acidity levels of samples from the Torul (Sample 3) and Kelkit (Sample 10) districts exceeded the established limits of 50 meq/kg by both the Turkish Food Codex and the Codex Alimentarius standards. Gürbüz et al^18^. reported free acidity values ranging from 2.00 to 44.00 meq/kg in 68 flower honey samples collected from the southeastern Anatolia region. Similarly, Bouhlali et al^26^. documented free acidity values ranging from 10.93 to 36.67 meq/kg in 11 Moroccan honeys of various floral origins. Differences in free acidity can be attributed to factors such as the presence of organic acids, floral origin, harvest season, and, to some extent, geographical conditions^7^. Furthermore, several studies have shown that free acidity in honey increases over time due to the fermentation of glucose and fructose by osmophilic yeasts. This fermentation produces alcohol and carbon dioxide. The alcohol can then oxidize into acetic acid in the presence of oxygen, which leads to increased acidity in honey^27^.

Proline, the predominant amino acid in honey, is added by bees during nectar processing^18^. In the present study, proline levels in the floral honey samples ranged from 562.66 mg/kg (Sample 14) to 1466.15 mg/kg (Sample 2). Statistically significant differences were observed among the samples (*p* < 0.05; Table 1). Bideci and Karasalihoğlu^30^reported that proline concentrations in honey samples collected from 40 producers in Kastamonu, Türkiye’s Black Sea region, between 2016 and 2019 ranged from 9.20 to 1176.10 mg/kg, with an average of 464.69 mg/kg. Similarly, Yetkin et al^28^. found proline contents ranging from 568 to 758 mg/kg in eight polyfloral and monofloral honey samples from various locations in Rize. All proline values recorded in the Gümüşhane samples exceeded the minimum threshold of 300 mg/kg established by the Turkish Food Codex Honey Communiqué, indicating their authenticity and quality.

The diastase activity of the honey samples in this study ranged from 25.00 to 60.00, with an average of 54.15 (Table 1). All of these values exceed the minimum requirement of 8 set by the Turkish Food Codex. These results suggest that the samples were not heat-treated and were stored under appropriate conditions, thus retaining their freshness. Yetkin et al^28^. found that the diastase activity of eight honey samples from the Cimil Plateau (Rize) ranged from 9.01 to 53.95. Their findings demonstrated that, while the average diastase activity remained above the legal threshold at 10 °C and 22 °C, values declined below 8 from the sixth month onward when stored at 35 °C, emphasizing the impact of storage temperature and duration. By contrast, Boutoub et al*.*^29^ reported a broader range of diastase activity in honeys of various floral origins, from 11.93 to 115.89. These comparisons support the stability and quality of the Gümüşhane honey samples analyzed in this study.

The HMF levels ranged from 0.37 to 18.89 mg/kg, with an average of 4.52 mg/kg (Table 1). All samples were well below the maximum limit of 40 mg/kg established by the Turkish Food Codex Honey Communiqué, suggesting that the honeys were fresh and had not undergone excessive heating. Gürbüz et al^18^. reported an average HMF value of 18.50 mg/kg in honey samples collected from various regions of Türkiye. Çelemli et al^31^. determined that the HMF content in a total of twenty honey samples produced in Ayder/Rize-Türkiye, including both monofloral and multifloral types, ranged from 0.70 mg/kg to 11.31 mg/kg, with an average of 3.80 mg/kg. In a comparable study, Živkov Baloš et al^25^. reported that the highest HMF level detected in sunflower honey was 4.41 mg/kg. The results align with earlier research and indicate that the HMF concentrations in the current samples remain within permissible levels.

### Sugar composition

In the present study, the total fructose and glucose contents of Gümüşhane honeys ranged from 60.58 to 78.99 g/100 g, while the sucrose content varied between 1.05 and 5.10 g/100 g, remaining within the limits established by the EU and Turkish Food Codex standards (Table 2). The F/G ratio (0.93–1.35) indicated that fructose was predominant in all samples, suggesting a lower crystallization tendency and confirming the natural floral origin of the honeys.

**Table 2:** Sugar content of different floral honeys.

Samples		Sugar analysis		
		Sucrose (g/100 g)	F + G (g/100 g)	F/G ratio
1		1.48 ± 0.63^a^	66.80 ± 1.41^ab^	1.18 ± 0.03^ab^
2	Torul	nd	66.63 ± 1.13^ab^	1.35 ± 0.03^c^
3		1.05 ± 0.43^a^	73.27 ± 1.03^c^	1.05 ± 0.04^a^
4		1.15 ± 0.17^a^	**78.99 ± 1.30**^** cd**^	0.93 ± 0.08^a^
5	Şiran	3.63 ± 0.44^c^	62.25 ± 1.30^a^	1.05 ± 0.06^a^
6		2.57 ± 0.55^b^	65.72 ± 1.17^ab^	1.00 ± 0.04^a^
7		nd	60.58 ± 1.16^a^	1.13 ± 0.04^a^
8	Kürtün	1.80 ± 0.68^a^	68.26 ± 1.09^ab^	1.22 ± 0.01^b^
9		1.56 ± 0.22^a^	63.82 ± 0.90^a^	0.97 ± 0.03^a^
10		1.90 ± 0.21^a^	72.13 ± 0.90^bc^	1.06 ± 0.03^a^
11	Kelkit	**5.10 ± 0.11**^**d**^	74.92 ± 1.10^b^	1.08 ± 0.05^a^
12		1.65 ± 0.37^a^	64.50 ± 0.73^ab^	1.25 ± 0.07^b^
13		2.20 ± 0.56^b^	72.53 ± 0.73^bc^	1.11 ± 0.04^a^
14	Köse	nd	75.04 ± 1.35^bc^	1.11 ± 0.08^a^
15		nd	68.85 ± 1.20^ab^	1.17 ± 0.03^ab^
*Overall Mean* ± *SD* Maximum Minimum		2.19 ± 1.21 5.18 0.74	68.95 ± 5.29 79.91 59.76	1.11 ± 0.11 1.37 0.89

*Mean values for 5 honey samples from each region. *F* Fructose, *G* Glucose.

^∗^*nd* Not determined. Bold values indicate the most pronounced results — highest sugar.

Baloš et al^23^. conducted comparative studies on various unifloral and multifloral honeys from Serbia and reported that sucrose content ranged from 0.25 to 2.86 g/100 g, F + G values ranged from 69.76 to 79.92 g/100 g in multifloral honeys, and F/G ratios varied from 0.93 to 1.42. Similarly, Bayram et al^22^. reported that sixty honey samples collected in Türkiye had sucrose levels ranging from 1.19 to 3.46 g/100 g, F + G contents ranging from 74.67 to 79.45 g/100 g, and F/G ratios ranging from 1.15 to 1.26. In the study conducted by Kara et al^32^. on Tokat honeys, the F + G content of the samples ranged from 62.54 to 76.67 g/100 g, while sucrose levels varied between 0.30 and 1.96 g/100 g. The fructose-to-glucose ratio (F/G) was calculated between 0.98 and 2.62. Likewise, Ferrassi et al^33^. reported that eight Moroccan monofloral honeys contained total reducing sugars between 77 and 81 g/100 g. The sucrose levels were below 0.10 g/100 g, and the F/G ratios ranged from 1.20 to 6.50. In the current study, significant variations (*p* < 0.05) were observed among samples in both F + G and F/G values. All values were within the acceptable limits established by the Codex Alimentarius and the Turkish Food Codex Honey Communiqué (F + G ≥ 60 g/100 g; sucrose ≤ 5 g/100 g). The results are consistent with findings from nearby regions such as Tokat and Bayburt^22,32^, indicating that the analyzed honeys were natural and properly matured. The F/G ratio close to 1 also suggests a low crystallization tendency, further confirming the good quality of the Gümüşhane honeys.

### Mineral composition

The average concentrations of Ca, Mg, Na, K, Fe, Zn, Cu, Al, Mn, Cd, Co, Ni, Pb, and Cr were determined in the honey samples and are presented in Table  3 and 4. The wide variation observed in the concentrations of these 14 elements highlights the mineral diversity of the samples. The most abundant mineral was potassium, followed by calcium, sodium, magnesium, iron, zinc, and copper. Among the toxic elements, Al showed the highest concentrations (0.46–73.51 mg/kg), followed by Mn (0.13–3.32 mg/kg), Ni (< 0.19–0.42 mg/kg), Pb (< 0.13–0.41 mg/kg), and Cr (< 0.15–0.29 mg/kg). Cadmium and cobalt were detected at the lowest levels, both being below 0.13 mg/kg. Samples 7, 8, and 9, all originating from the Kürtün district, exhibited the highest concentrations of all measured minerals among the analyzed honey samples.

**Table 3:** Major elements/trace elements of honey samples (mg/kg).

Samples		Ca	Mg	Na	K	Fe	Zn	Cu
1		26.50±1.30^bc^	9.40±0.40^c^	22.10±0.50^b^	342.00±16.20^bc^	3.80±0.10^ab^	0.58±0.02^a^	1.02±0.04^c^
2	Torul	14.70±0.69^ab^	10.00±0.41^c^	34.90±2.10^c^	195.40±10.20^ab^	3.20±0.10^a^	0.37±0.02^a^	1.48±0.05^d^
3		35.10±1.99^c^	8.80±0.17^b^	32.30±1.20^c^	462.2±19.50^c^	3.30±0.10^a^	1.99±0.07^b^	0.68±0.03^b^
4		10.0±0.27^a^	4.40±0.98^a^	7.20±0.30^a^	159.90±6.80^ab^	3.50±0.20^a^	0.49±0.01^a^	0.63±0.02^b^
5	Şiran	21.10±0.80^ab^	8.10 ±0.29^ab^	3.40±0.99^a^	98.20±3.90^a^	6.30±0.20^c^	<0.23	<0.15
6		36.00±2.19^c^	11.50±0.60^cd^	7.50±0.30^a^	342.60±17.09^bc^	5.30±0.20^bc^	0.78±0.04^a^	0.27±0.02^a^
7		168.50±7.17^ı^	51.80±2.80^f^	53.50±2.20^d^	3453.40±162.21^g^	19.50±1.00^f^	0.64±0.04^a^	2.98±0.12^f^
8	Kürtün	130.90±4.29^h^	42.25±2.00^e^	54.20±2.70^d^	1951.20±66.00^f^	16.60±0.70^e^	1.90±0.08^b^	2.25±0.12^e^
9		89.40±3.70^f^	43.10±1.90^e^	126.50±5.70^f^	966.00±19.40^d^	8.60±0.40^d^	15.39±0.71^d^	1.39±0.06^d^
10		39.20±2.10^d^	11.00±0.41^c^	28.90±1.20^bc^	225.10± 8.60^ab^	5.10±0.10^bc^	0.82±0.05^a^	0.97±0.02^c^
11	Kelkit	101.59±4.25^g^	18.50±0.80^d^	58.60± 2.20^de^	1232.30±63.40^e^	9.20±0.40^d^	0.73±0.04^a^	1.00±0.02^c^
12		79.90±2.98^f^	15.40±0.60^cd^	37.10±1.40^c^	402.90±19.80^c^	3.40±0.10^a^	11.13±0.47^c^	0.62±0.03^b^
13		51.50±3.28^d^	14.00±0.51^c^	66.30±2.10^e^	430.30±12.40^c^	4.50±0.10^ab^	2.64±0.08^b^	0.32±0.02^a^
14	Köse	14.20±0.57^ab^	4.60±0.30^a^	10.40±0.40^a^	110.10±4.10^a^	6.50±0.30^c^	<0.23	0.17±0.03^a^
15		67.29±3.16^e^	9.60±0.30^c^	22.70±0.40^b^	193.70±5.90^ab^	9.50±0.40^d^	<0.23	1.35±0.05^d^
*Overall Mean ± SD*		59.06±46.15	17.50±14.94	37.71±31.12	704.35±899.45	7.22±4.84	2.53±4.41	1.02±0.78
Maximum Minimum		173.57	53.78	130.53	3568.10	20.20	15.89	3.06
		9.81	4.33	3.33	95.44	3.13	0.17	0.10

Different letters within the same column indicate significant differences (*p* < 0.05) based on one-way ANOVA followed by Tukey’s HSD test.

*SD Standard deviation.

Bold values indicate the highest concentrations for each element.

Potassium levels in the analyzed honey samples were higher than those reported in honeys from Morocco (263.40–1487.71 mg/kg)^26^ and the Punjab region of Pakistan (166–1732 mg/kg)^34^. The highest Ca concentration was detected in Sample 7 at 168.50 mg/kg. This result aligns with the range reported by Živkov Baloš et al^25^., which varied from 70.93 to 152.72 mg/kg. Sodium was identified as the third most abundant mineral element, with concentrations varying significantly between 3.40 and 126.50 mg/kg (Table 3). Similar variability has been reported by Solayman et al^24^., who documented Na concentrations ranging from 3.23 to 236.80 mg/kg in honeys of various botanical origins, and by Boutoub et al^29^., who reported a range of 34.96 to 125.05 mg/kg in Euphorbia honeys. The average Mg content in the analyzed honey samples was determined to be 17.50 mg/kg, with statistically significant differences observed among the samples. This value is consistent with the Mg content reported in some natural honeys produced in Türkiye, which ranged from 5.00 to 34.00 mg/kg^35^. The major elements identified in the honey samples were K, Na, Ca, and Mg, which confirms trends observed in previous studies^26,36^. However, the relatively high potassium and calcium levels detected in Gümüşhane honeys were greater than those reported for both domestic (e.g., Tokat and Bayburt)^22,32^ and international honeys (e.g., Morocco and Pakistan)^26,34^. This enrichment likely reflects the region’s mineral-rich soils and mountainous vegetation, which contribute to higher mineral uptake from nectar sources, distinguishing Gümüşhane honeys from other regional and global varieties.

**Table 4:** Major elements/trace elements of honey samples (mg/kg).

Samples		Al	Mn	Cd	Co	Ni	Pb	Cr
1		6.54±0.18^c^	0.22±0.02^ab^	<0.13	<0.13	<0.19^a^	0.16 ± 0.01^ab^	<0.15
2	Torul	6.33±0.24^c^	0.24±0.02^ab^	<0.13	<0.13	<0.19^a^	0.14 ± 0.01^ab^	<0.15
3		7.04±0.21^c^	0.18±0.02^ab^	<0.13	<0.13	0.28 ± 0.02^b^	0.21 ± 0.01^b^	<0.15
4		0.46±0.01^a^	0.18±0.02^ab^	<0.13	<0.13	<0.19^a^	0.15 ± 0.01^ab^	<0.15
5	Şiran	6.08±0.23^c^	0.34±0.02^bc^	<0.13	<0.13	0.29±0.02^b^	0.20 ± 0.01^b^	<0.15
6		3.94±0.20^bc^	0.28±0.01^ab^	<0.13	<0.13	<0.19^a^	<0.13^a^	<0.15
7		72.94±1.50^f^	3.32±0.11^g^	<0.13	<0.13	0.42 ± 0.03^c^	0.41 ± 0.03^e^	0.29 ± 0.02^c^
8	Kürtün	73.51±2.54^f^	2.45±0.10^f^	<0.13	<0.13	<0.19^a^	0.31 ± 0.02^d^	<0.15
9		28.31±1.30^e^	2.04±0.07^e^	<0.13	<0.13	0.31 ± 0.02^b^	0.21 ± 0.02^b^	<0.15
10	Kelkit	5.93±0.21^bc^	0.19±0.01^ab^	<0.13	<0.13	<0.19^a^	0.21 ± 0.02^b^	<0.15
11		18.68±0.68^d^	0.86±0.04^d^	<0.13	<0.13	<0.19^a^	0.17 ± 0.01^ab^	0.20 ± 0.02^b^
12		5.43±0.20^bc^	0.14±0.01^a^	<0.13	<0.13	<0.19^a^	<0.13^a^	<0.15
13		4.28±0.14^bc^	0.44±0.02^c^	<0.13	<0.13	<0.19^a^	<0.13^a^	<0.15
14	Köse	2.55±0.09^ab^	0.13±0.01^a^	<0.13	<0.13	<0.19^a^	0.20 ± 0.01^b^	<0.15
15		6.79±0.25^c^	0.16±0.01^ab^	<0.13	<0.13	<0.19^a^	0.24 ± 0.02^c^	<0.15
*Overall Mean ± SD* Maximum Minimum		16.58±23.59	0.74±0.99	0.13±0.00	0.13±0.00	0.20±0.08	0.20±0.08	0.15±0.04
		75.31 0.45	3.40 0.12	0.13 0.13	0.13 0.13	0.44 0.14	0.43 0.10	0.30 0.12

Different letters within the same column indicate significant differences (*p* < 0.05) based on one-way ANOVA followed by Tukey’s HSD test.

*SD Standard deviation.

Bold values indicate the highest concentrations for each element.

The concentrations of iron, copper, and zinc in the honey samples ranged from 3.20 to 19.50 mg/kg for Fe, < 0.15 to 2.98 mg/kg for Cu, and < 0.23 to 15.39 mg/kg for Zn. Tutun et al^37^. reported that Cu levels in honeys from Isparta, Antalya, and Burdur ranged between 0.04 and 1.52 mg/kg, while Fe and Zn concentrations varied from 0.20 to 16.36 mg/kg and 0.32 to 6.00 mg/kg, respectively. These findings are consistent with the Fe, Cu, and Zn levels observed in the 15 honey samples analyzed in the present study. The Al concentrations in the analyzed honey samples ranged from 0.46 to 73.51 mg/kg. These values are partly comparable to those reported in 12 honey samples produced in the northern region of Malaysia by Jamaludin et al^38^. (25.35–662.25 mg/kg), as well as to the values reported by Lobos et al^36^. for honeys produced in the central–southern region of Chile (27.50–47.80 mg/kg). However, two samples (Sample 7: 72.94 mg/kg and Sample 8: 73.51 mg/kg) exhibited notably higher Al levels than the ranges reported in these studies (Table 4). Elevated Al levels may result from the use of aluminum-based equipment in beekeeping practices or from environmental contamination associated with agricultural or mining activities^24^. Furthermore, the mean Al concentration observed in this study (16.58 mg/kg) was higher than the values previously reported for certain regional Turkish honeys, including multifloral honey (5.36 mg/kg)^39^ and highland honeys (7.20 mg/kg)^20^. These differences likely reflect the influence of local geological structure, soil composition, and potential anthropogenic inputs in the honey production areas^22,32^. The relatively high Al concentrations observed in some samples may be associated with the regional geological structure of Gümüşhane, which is rich in aluminosilicate minerals and affected by mining activities^40^. In addition to potential anthropogenic inputs, the region’s acidic soil conditions and high precipitation may enhance aluminum mobility, facilitating its transfer from soil to plant nectar and consequently to honey^41^.

Heavy metals such as Cd, Pb, Ni, Co, and Cr may contaminate bee colonies and their products through environmental exposure, particularly from industrial emissions and traffic-related pollution. These elements can enter the food chain via air, water, and soil, ultimately reaching nectar and honey^42^. The lead concentrations in the analyzed samples (< 0.13–0.41 mg/kg) are comparable to those reported in Polish multifloral (0.02–1.01 mg/kg) and monofloral (0.01–0.34 mg/kg) honeys^43^. The overall heavy metal levels observed in this study are also consistent with those reported in highland honeys produced in Northeastern Türkiye, with concentrations of Cd: 0.13; Cr: 0.33; Pb: 0.18; and Ni: 0.14 mg/kg^20^. Similarly, the Ni concentrations in the current samples (< 0.19–0.42 mg/kg) align with the range reported for honeys from the Tokat region (≤ 2.91 mg/kg)^32^. Overall, the heavy metal concentrations measured in the samples were low and within the ranges previously reported for uncontaminated honeys; however, no quantitative human health risk assessment (e.g., THQ or HI) was conducted in this study.

Heavy metals such as Cd, Pb, Ni, Co, and Cr may contaminate bee colonies and their products through environmental exposure, particularly from industrial emissions and traffic-related pollution. These elements can enter the food chain via air, water, and soil, ultimately reaching nectar and honey^42^. The overall heavy metal levels observed in this study are consistent with those reported in highland honeys from Northeastern Türkiye, with concentrations of Cd: 0.13; Cr: 0.33; Pb: 0.18; and Ni: 0.14 mg/kg^20^. The lead concentrations in the analyzed samples (< 0.13–0.41 mg/kg) were also comparable to those reported in Polish multifloral (0.02–1.01 mg/kg) and monofloral (0.01–0.34 mg/kg) honeys^43^. Furthermore, Sahinler et al^39^. reported Cd (0.30 mg/kg), Cr (0.46 mg/kg), Pb (0.82 mg/kg), Ni (0.48 mg/kg), and Co (0.06 mg/kg) in multifloral honeys collected from different regions of Türkiye, although concentrations varied among botanical origins such as citrus, cotton, sunflower, and chestnut. In the present study, the highest Pb concentration (0.41 mg/kg) was detected in Sample 7 from the Kürtün district, which also exhibited relatively elevated Ni (0.42 mg/kg) and Cr (0.29 mg/kg) levels, suggesting localized environmental influence on metal accumulation. Overall, the heavy metal concentrations measured in the samples were comparable to those reported in previous studies on honeys from Türkiye and other regions; however, no health risk assessment (e.g., THQ or HI) was performed in this study. Although direct comparison between honey and pollen is not appropriate due to matrix differences, a recent study from the Eastern Black Sea region reported relatively high Pb, Cd, and Ni levels in bee pollen, suggesting that environmental factors such as soil composition and agricultural activities may influence the presence of these elements in bee products in this region^44^. Additionally, the relatively high levels of Al, Ni, Pb, and Cr observed in some honey samples may be related to the regional geology of Gümüşhane, characterized by antimonite-rich mineralization zones and hydrothermal alteration. Sungur et al^45^. reported increased Cu, Zn, Ni, and Pb levels in nearby agricultural soils due to these processes, with metals showing high mobility in non-residual fractions. Such geological conditions may facilitate the transfer of metals from soil to plants and nectar, contributing to the elevated levels detected in certain honey samples.

## Antioxidant potential of honey

**Fig. 1:**
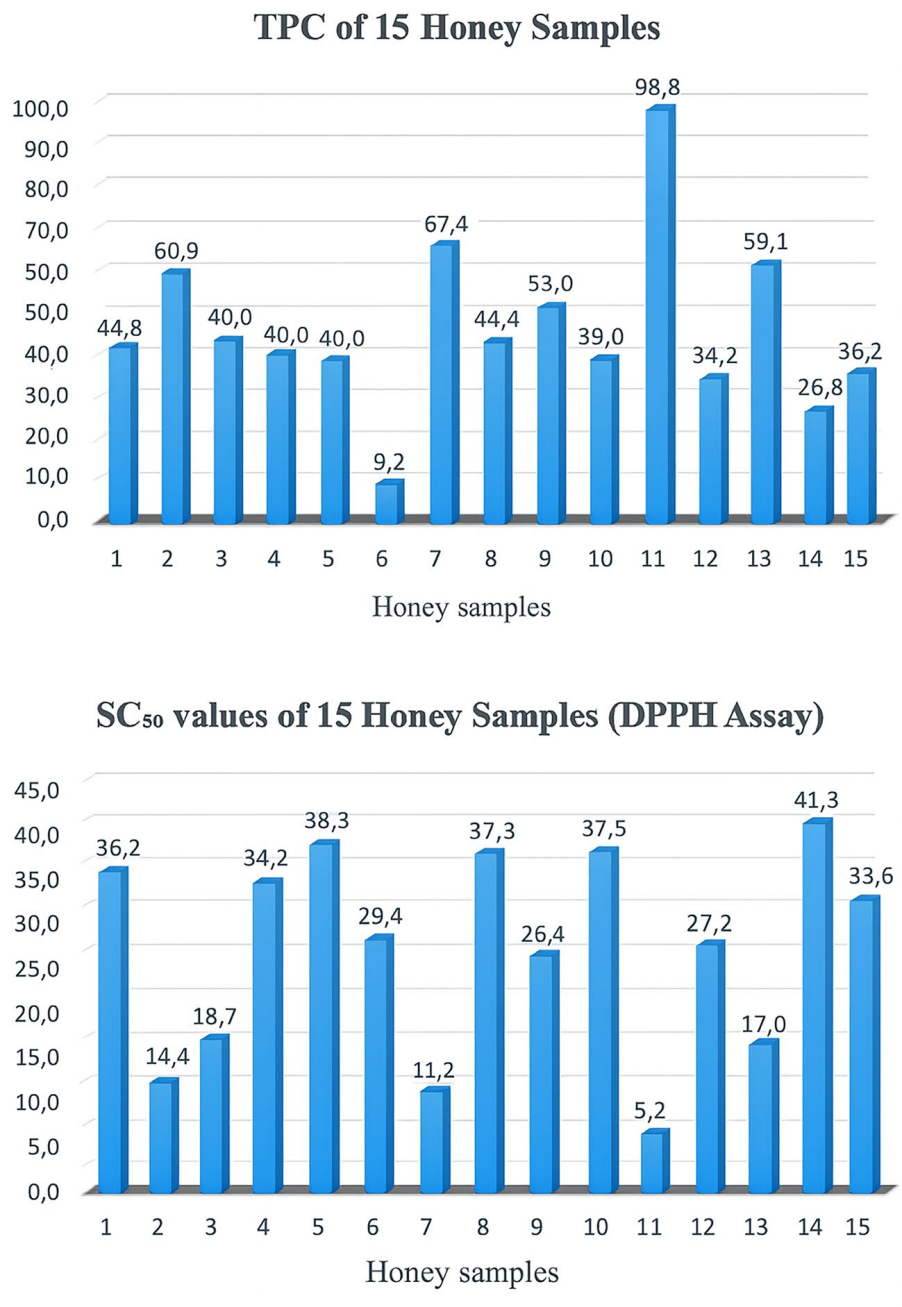
Total phenolic content (mg GAE/100 g) and SC_50_ values (mg/mL) of the 15 honey samples.

The total phenolic contents of the honey samples are presented in Fig. 1. Based on the gallic acid calibration curve (R^2^= 0.999), the total phenolic content of the samples ranged from 9.21 to 98.25 mg GAE/100 g, with statistically significant differences observed among them (*p* < 0.05). The phenolic composition of various honey extracts has also been reported in previous studies. Ekici et al^46^. determined the total phenolic content of 20 pine honey samples from the Muğla and Marmaris regions of Türkiye to be 35.30–92.87 mg GAE/100 g. Can et al^47^. reported values of 29.54 mg/100 g in multifloral honeys and 16.02 mg GAE/100 g in acacia honeys. Similarly, Zawawi et al^48^. found that a multifloral honey sample from Brazil exhibited the highest phenolic concentration (78.20 mg GAE/100 g). Akgün et al^49^. reported that multifloral, chestnut, acacia, and rhododendron honeys contained phenolic levels ranging between 0.15–0.36 mg GAE/g, 0.09–0.16 mg GAE/g, 0.01–0.03 mg GAE/g, and 0.11–0.32 mg GAE/g, respectively. Kara et al^50^. reported that multifloral honeys produced in the Tokat region of Türkiye contained 85.54–148.26 mg GAE/100 g, indicating notably higher phenolic concentrations compared with many other regional honeys. Additionally, Karlıdağ^51^ reported total phenolic contents of 19.47–29.33 mg GAE/100 g in multifloral honeys from the Eastern Anatolia Region of Türkiye. Overall, the phenolic content range found in the present study is consistent with previously reported values for honeys from different botanical and geographical origins.

The radical scavenging activity of the honey samples was assessed using the DPPH assay, and the results were expressed as SC₅₀ (mg/mL), defined as the sample concentration required to inhibit 50% of the DPPH radical^52^. In the present study, SC₅₀ values of the 15 Gümüşhane honey samples ranged from 5.23 to 41.29 mg/mL, indicating notable variation in antioxidant capacity among the samples (*p* < 0.05). Karlıdağ et al^51^. reported SC₅₀ values between 36.71 and 46.63 mg/mL for multifloral honeys from Eastern Türkiye, while Kara et al^50^. found SC₅₀ values ranging from 34.83 to 78.67 mg/mL in honeys from the Tokat region. Can et al^47^. documented a broader range (12.56–152.40 mg/mL) in Turkish honeys of various floral origins, and Ertürk et al^53^. reported even wider variability (29.38–458.45 mg/mL) in honeys from the Eastern Black Sea region. International studies also demonstrate substantial variation; for example, Paul et al^54^. reported SC₅₀ values between 0.32 and 24.28 mg/mL in Bangladeshi honeys. In Brazil, Zawawi et al^48^. reported the lowest IC₅₀ value (10.81 mg/mL) in the multifloral honey sample with the highest phenolic content (78.20 mg GAE/100 g). Consistent with this pattern, the sample with the lowest SC₅₀ value in the present study also exhibited the highest total phenolic content (98.27 mg GAE/100 g). Overall, these findings reinforce previous reports demonstrating a strong association between total phenolic content and DPPH radical scavenging activity in honey.

## Materials and methods

### Chemicals

The chemicals used are, proline (CAS No: 147–85-3), Hydroxymethylfurfural (CAS No: 67–47-0), formic acid (CAS No: 64–18-6), ninhydrin (CAS No: 485–47-2), sucrose (CAS No: 57–50-1, glucose (CAS No: 50–99-7), fructose (CAS No: 57–48-7), potassium ferrocyanide (CAS No: 14459–95-1), zinc acetate dihydrate (CAS No: 5970–45-6), nitric acid (CAS No: 7697–37-2), gallic acid (CAS No: 149–91-7), and quercetin (CAS No: 117–39-5) were purchased from Merck Co. (Merck KGaA, Darmstadt, Germany).

## Honey samples

**Fig. 2:**
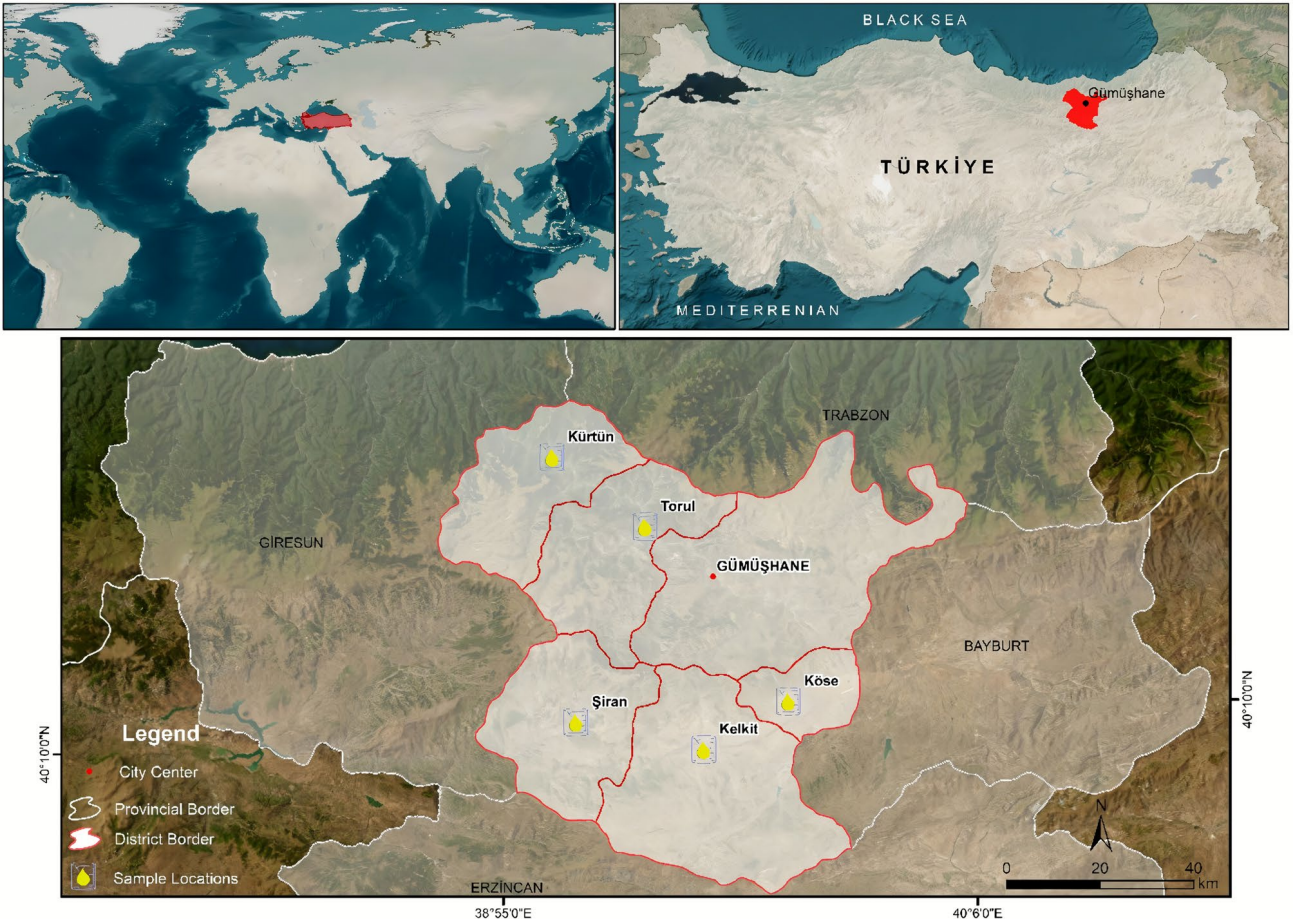
Location map of the sampling sites in Gümüşhane, Türkiye (The map was created using ArcGIS for Desktop Advanced 10.x (Esri, Redlands, CA, USA)).

A total of 15 natural honey samples were collected from local producers during the 2021 harvest season (August–September). According to the information provided by local beekeepers, no artificial feeding was applied to the colonies during the production season. This information was based on beekeeper declarations. The samples were from five distinct regions in Gümüşhane Province: Torul, Şiran, Kürtün, Kelkit, and Köse. The location map in Fig. 2 was generated by the authors using ArcGIS for Desktop Advanced 10.x (Esri, Redlands, CA, USA; https://www.esri.com). Base maps were used to digitize country, province, and district boundaries in vector format. Point features representing sampling locations were produced based on field observations and recorded in the geodatabase. The final location map was generated in the ArcMap interface. The map is original and has not been modified from any copyrighted source.

The 15 honey samples, each weighing 500 g, were stored in sterile glass jars at 18 °C in a dry and dark environment until analysis. Prior to analysis, each honey sample was homogenized to ensure consistency.

## Physicochemical analysis of honey samples

Moisture content was measured using an Atago PAL-BX/RI refractometer (Tokyo, Japan). Refractive index measurements were taken at 25 °C and corrected to 20 °C^23^. Final values were expressed as moisture percentage.

Conductivity was determined at 20 °C using a HANNA HI8819 meter, with 20 mg of honey diluted to 100 mL in distilled water^22^. The probe was inserted, and readings were recorded.

Free acidity was determined based on a modified version of the Bouhlali et al^26^. method. A 10 g honey sample was diluted in 75 mL of water and titrated with 0.1 N NaOH to pH 8.50.

Water-insoluble matter was measured following the method of Živkov Baloš et al*.*^25^. A specific amount of honey was dissolved in water at 80 °C and filtered through a sintered glass crucible with a defined pore size. The residue remaining on the crucible was washed with hot water, then dried and weighed. Water-insoluble content was quantified by weighing the dried residue remaining after filtration.

Following the protocol of Yetkin et al^28^., diastase activity was determined using a reaction mixture containing honey diluted with acetate buffer, water, and NaCl. A starch solution served as the substrate, and absorbance was recorded at 620 nm via spectrophotometry.

The proline content was detected using the method described by Bouhlali et al^26^. First, a 0.5 mL honey solution was mixed with 1 mL of ninhydrin reagent (3 g dissolved in 100 mL of ethylene glycol monomethyl ether) and 1 mL of formic acid (80%). The sample was heated in boiling water for 15 min, then incubated at 70 °C for 10 min. After adding a 50% isopropanol solution (5 mL), the mixture was cooled for 45 min. Absorbance was measured at 510 nm. Deionized water served as the blank, while a 0.032 mg/mL proline solution functioned as the reference. The proline content was expressed in mg/kg, based on the corresponding calibration formula.

1 $$\Pr oline(mg/kg) = (E_{s} /E_{a} ) \times \left( {E_{w1} /E_{w2} } \right) \times 80$$

Here, *Es* denotes the optical density measured for the sample solution, while *Ea* refers to the average absorbance obtained from the proline reference. *Ew1* indicates the mass of proline present in the standard solution (mg), *Ew2* corresponds to the weight of the honey sample (g), and the constant 80 reflects the applied dilution factor.

## Sugar analysis

The sugar profile (glucose, fructose, and sucrose) was analyzed based on the method of Yuksel et al*.*^55^. Honey samples (5 g) were extracted with water and methanol, filtered (0.45 μm), and analyzed using HPLC-RID equipped with a carbohydrate column. Separation was performed at 30 °C with an acetonitrile/water (80:20) mobile phase at 1.3 mL/min. Quantification was based on calibration curves, and results were expressed as fructose-to-glucose ratio (F/G), total invert sugar, and sucrose content.

## HMF analysis

HMF levels were analyzed using the method of Yuksel et al^55^. with an HPLC system (Thermo Finnigan, USA) equipped with a UV detector set at 285 nm. Honey samples (5 g) were clarified using Carrez reagents, passed through a 0.45 μm filter, and applied to a C18 reversed-phase column. The chromatographic separation was performed using a 90:10 (v/v) mixture of water and methanol at a flow rate of 1 mL/min. Concentrations were determined using a standard curve generated from HMF solutions in the range of 1.0 to 10.0 mg/L and reported in mg/kg.

## Elemental analysis

Following the method of Boutoub et al^29^., 5 g of each honey sample was incinerated in porcelain crucibles at 550 °C in a muffle furnace until a white ash residue was obtained. The ash obtained from the furnace was dissolved with 10% nitric acid and the volume was made up to 50 mL with distilled water. To determine the trace elements and heavy metals (K, Ca, Na, Mg, Zn, Fe, Cu, Al, Mn, Pb, Cd, Co, Cr, Ni) in the samples, an Agilent Technologies (Santa Clara, California, USA) 4200 model MP-AES (Microwave Plasma Atomic Emission Spectrometer) was used. Prior to measurements, a series of standard solutions was prepared by diluting certified single-element stock solutions (1000 mg/L) with appropriate ratios (0.025–10.0 mg/L). These standard solutions were measured using the MP-AES to derive calibration curves for the relevant metals. Subsequently, the dissolved sample solutions were analyzed with the device to obtain signal values for each metal. The concentrations of each metal were determined using linear Eq. (2) derived from the calibration curves (*S* = *mC* + *n*, where *S* is the signal, *C* is the concentration, *m* is the slope, and *n* is the intercept). The concentrations were then converted to ppm (mg/kg) using the following formula*:*

2 $$Concentration\left( {mg/kg} \right) = CxVxS/m$$

where, *C* represents the concentration measured in mg/L by MP-AES; *V* is the final volume of the sample (mL); *m* is the mass of the weighed sample (g) and *S* is the dilution factor.

## Bioactivity analysis

### Determination of TPC

TPC was measured using a modified version of the method by Bayram et al^22^. For the analysis, 300 µL of honey sample was combined with 3.4 mL distilled water, 0.5 mL methanol, and 200 µL Folin-Ciocalteu reagent. The reaction proceeded for 10 min at room temperature. Then, 600 µL of 10% sodium carbonate (Na₂CO₃) solution was added, vortexed again, and incubated in the dark for 2 h. Readings were taken at 760 nm using a spectrophotometer. A blank solution containing all reagents except honey was used as reference. TPC was calculated using a calibration curve prepared with gallic acid (31.25–500 mg/L, R^2^ = 0.99) and expressed as milligrams of gallic acid equivalents per 100 g of honey.

### Analysis of antioxidant activities

The DPPH assay, with modifications from the method of Ertürk et al^42^., was employed to evaluate the radical scavenging capacity of the honey samples. A solution was prepared by dissolving 2 g of honey in 10 mL of distilled water. An equal volume of 0.1 mM DPPH in methanol was added, and the mixture was kept in the dark for 50 min at room temperature. Absorbance was then measured at 517 nm. Trolox standards (0.1–100 µg/mL) were used for calibration, and results were expressed as SC₅₀ (µg/g).

## Statistical analysis

Data were analyzed using IBM SPSS 27. All measurements were performed in triplicate, and the results were evaluated by one-way ANOVA followed by Tukey’s test (*p* < 0.05).

## Conclusions

This study examined the physicochemical characteristics, mineral content, and antioxidant capacity of 15 honey samples sourced from various districts of Gümüşhane, Türkiye. The moisture content, electrical conductivity, and free acidity values largely complied with the Turkish Food Codex, indicating good beekeeping practices. Notably, the proline content exceeded the required minimum, while HMF levels remained low, underscoring the freshness and quality of the samples. The mineral composition of the honeys was both qualitatively and quantitatively rich, with major elements including potassium, calcium, magnesium, and sodium. The relatively high potassium and calcium concentrations reflect the influence of the region’s mineral-rich soils and mountainous flora, while higher aluminum levels in some samples may be linked to local geological and environmental factors. The antioxidant activity (SC₅₀) also demonstrated strong bioactivity compared to other regional honeys.

Overall, these findings confirm the high quality of Gümüşhane honeys and highlight their potential as a distinctive regional product with nutritional and commercial value. Future studies should include multi-year sampling, melissopalynological verification of floral origin, and additional bioactivity assays (e.g., antimicrobial and anti-inflammatory analyses) to further establish the functional and geographical identity of Gümüşhane honeys.

## Data Availability

All data generated or analyzed during this study are included in this article.
